# Emotion Dysregulation as a Mediator of the Relationship between Anxiety, Compulsive Exercise and Eating Disorder Symptoms in Adolescents

**DOI:** 10.3390/children8121088

**Published:** 2021-11-25

**Authors:** Cristina Cuesta-Zamora, Irene González-Martí, Luis Miguel García-López, Laura Ros, Carolyn R. Plateau, Jorge Javier Ricarte

**Affiliations:** 1 Psychology Department, Faculty of Education, University of Castilla-La Mancha, 02071 Albacete, Spain; cristina.cuesta@uclm.es; 2 Music, Arts Education and Physical Education Department, Faculty of Education, University of Castilla-La Mancha, 02071 Albacete, Spain; irene.gmarti@uclm.es (I.G.-M.); luismiguel.garcia@uclm.es (L.M.G.-L.); 3 Psychology Department, Faculty of Medicine, University of Castilla-La Mancha, 02006 Albacete, Spain; laura.ros@uclm.es; 4 National Centre for Sport and Exercise Medicine, School of Sport, Exercise and Health Sciences, Loughborough University, Leicestershire LE11 3TU, UK; C.R.Plateau@lboro.ac.uk

**Keywords:** compulsive exercise, rumination, eating disorder symptoms, emotional suppression, adolescents

## Abstract

Anxiety has been suggested to be a key contributing factor for compulsive exercise, however, existing literature has demonstrated contradictory relationships between anxiety and compulsive exercise among adolescents. According to the Emotional Cascade Model (ECM), factors such as rumination and emotional suppression may mediate the association between affect and exercise. The current study therefore aimed to investigate whether rumination and emotional suppression mediate the relationship between anxiety and compulsive exercise in predicting ED symptoms in adolescents. Questionnaires assessing compulsive exercise, anxiety, depressive rumination, emotional suppression, and ED symptoms were completed by 212 adolescent males (Mage = 13.39, SD = 1.22) and 189 adolescent females (Mage = 13.64, SD = 1.29). The structural equation model showed indirect effects between anxiety and compulsive exercise through rumination and emotional suppression in males but not in females. Moreover, anxiety had an indirect effect on eating disorder symptoms through rumination, emotional suppression and compulsive exercise in both males and females. In line with ECM, the results suggest that rumination and emotional suppression may have a key role in the association between anxiety, compulsive exercise and eating disorder symptoms in adolescents. These findings suggest that compulsive exercise may be used as a dysfunctional coping mechanism to escape from a negative emotional cascade generated by the interaction of anxiety, rumination and emotional suppression. Future longitudinal studies to test the role of compulsive exercise as a dysfunctional behaviour in the ECM are needed.

## 1. Introduction

Compulsive exercise is a multidimensional construct, whereby exercise attitudes and behaviours are characterised by weight and shape concerns, and persistent continuation of exercise in order to mitigate the experience of extreme guilt and/or negative affect when unable to exercise, and to avoid the perceived negative consequences of stopping [1]. Compulsive exercise has been identified as an aetiological and maintaining factor of eating disorder (ED) psychopathology [1,2,3], and has been associated with impaired quality of life [4,5], greater psychological distress [4,5], higher risk of suicidal behaviour [6,7,8], and higher levels of substance use [8,9] in both clinical and general populations.

However, despite the clinical significance of compulsive exercise, it remains understudied compared to other psychopathological aspects of EDs [10]. In particular, the role of anxiety in the aetiology and maintenance of compulsive exercise [1], remains unclear in adolescent both with and without EDs [11]. The compulsive exercise model [1] proposes that exercise could be maintained via positive reinforcement mechanisms (due to the mood enhancing effects of exercise) and via a negative reinforcement mechanism (i.e., where exercise is conducted to avoid or mitigate the experience of guilt and/or negative affect). However, in adolescents, evidence of the relationship between compulsive exercise and affect is contradictory. In clinical populations, while some studies showed a significant positive association between compulsive exercise and anxiety [12], other research did not find associations between both constructs [13]. In non-clinical samples, for example, McCabe and Ricciardelli [14] reported positive relationships between exercise dependence and negative affect, whereas Goodwin and colleagues [15] reported that neither anxiety nor depression could predict compulsive exercise. Further, lower anxiety levels were found to be predictive of compulsive exercise symptoms at two-year follow-up in males [16].

The Emotional Cascade Model (ECM) [17] proposes that cognitive emotion regulation strategies (i.e., emotional suppression and rumination) may be linked to dysfunctional behaviours (such as self-injury and drinking to cope). According to the ECM, the association between emotional suppression and rumination on negative affect could increase the magnitude of that negative affect. Therefore, in order to distract from this negative affect and rumination, individuals may use dysfunctional behaviours. Previous explorations of the ECM have shown validity in explaining the occurrence of behaviours such as self-harm [17,18], drinking to cope [17] in both cross-sectional and longitudinal studies [17,19,20]. Within the eating disorder field, the ECM has also shown validity in the prediction of bulimic symptoms [20], suggesting that disordered eating behaviours may be used as a way to reduce negative affect and rumination. However, although compulsive exercise is a key element of eating disorders, this has yet to be explored in the ECM.

One key aspect of the emotional cascade process is rumination. Rumination is defined as the tendency to repeatedly focus on the experience of negative emotion, as well as the causes and consequences of that negative emotion [21]. In the ECM, being involved in rumination may increase negative affect, which in turn, may increase negative thoughts in a vicious, repetitive cycle, i.e., an emotional cascade [17]. In relation to EDs, a growing body of literature suggests that individuals who ruminate might be in a greater risk of body dissatisfaction [22] and eating disorder psychopathology [23,24]. Specifically, in the female adolescent population, rumination has been associated with greater loss of control over eating [25] and bulimic symptoms [26]. Moreover, Arbuthnott et al. [19] found that individuals with a history of eating disorder behaviours had a greater decrease in positive emotions after a rumination induction compared to those without a history of eating disorder behaviours.

In addition, evidence analysing the link between rumination and other ED-related factors (such as compulsive exercise) is still in its infancy. Rumination has been proposed as an aetiological and maintenance factor of two key pillars of compulsive exercise: negative affect [27] and ED symptoms [28,29]. Indeed, rumination is clearly linked with the other two key components of compulsive exercise: perfectionism [30] and obsessive-compulsiveness [31]. If rumination is related to all key components of compulsive exercise, it is plausible to suggest that it will have a key role in its aetiology and/or maintenance. To our knowledge, there is just one study to date that has focused on examined the relationship between rumination and excessive exercise in university students [32], but significant associations between the constructs were not demonstrated for either males or females. However, due to the differences in ED symptoms between adults and adolescents [33], further research is needed in adolescents.

Emotional suppression refers to the ongoing inhibition of the expression of emotions [34]. Literature suggests that emotional suppression may increase negative emotions [17]. Emotional suppression is common among clinical ED populations [35] and individuals at risk of EDs [36]. Emotional suppression has been associated with increased body dissatisfaction [35,37] and ED symptoms [36]. However, as far as we are aware, the relationship between exercise and emotional suppression has only been studied in women with Bulimia Nervosa (BN) [38] and in university students [32], which demonstrated contradictory results. Brownstone et al. [38] showed that individuals with higher levels of affective lability and lower levels of emotional expression showed tendencies towards higher levels of compulsive exercise. However, thought suppression, which is defined as the tendency to suppress unwanted or negative thoughts [39], was found not to be associated with excessive exercise [32].

Whilst in university student populations rumination and thought suppression have not been associated with excessive exercise [32], in adolescent populations emotional dysfunctional strategies have been identified as cross-sectional [40] and longitudinal [41] predictors of compulsive exercise. However, in these studies [40,41], dysfunctional strategies (specifically, self-harm, rumination and repression) were evaluated together, hence, the specific role of each emotional regulation strategy in the development and maintenance of compulsive exercise could not be identified. A possible explanation for the discrepancy of results between adolescent and university samples could be that dysfunctional emotion regulation strategies could be differentially related to ED symptoms [32]. Moreover, the role of anxiety in the aetiology of compulsive exercise was not explored in Goodwin et al. [40,41] studies. Therefore, further research is needed to clarify and understand the complex associations between anxiety, emotion regulation strategies, compulsive exercise and ED symptoms [41].

A growing body of literature suggests sex differences in rumination, emotional suppression and compulsive exercise in adolescents. Whilst it is acknowledged that males tend to ruminate less than females [42], results have shown that rumination has been positively associated with body dissatisfaction and ED symptoms in both males [25] and females [22]. However, evidence suggests that rumination could have a different impact on body dissatisfaction [22] and on eating pathology [43] in males and females. Regarding emotional suppression, some studies suggest that it may be less dysfunctional in males than in females [44]. For example, in an experimental study in which participants were encouraged to attend to or suppress their emotional responses to negative pictures, males were identified as having more adaptive psychological consequences than females in the emotional suppression condition [44]. Neurocognitive experimental studies with MRI scanning techniques have also demonstrated gender differences in the use of emotional suppression [45]. Finally, regarding compulsive exercise, previous research suggests that males with EDs tend to show higher levels of excessive exercise [46], overactivity [47] and more harmful cognitions and attitudes towards exercise [48,49] compared to females with EDs. Moreover, exercise is the only compensatory behaviour that could be employed not only for weight reduction, but also to increase weight and muscle size [50]. Thus, examining the ECM in males and females separately may help to clarify the results.

**Figure 1:**
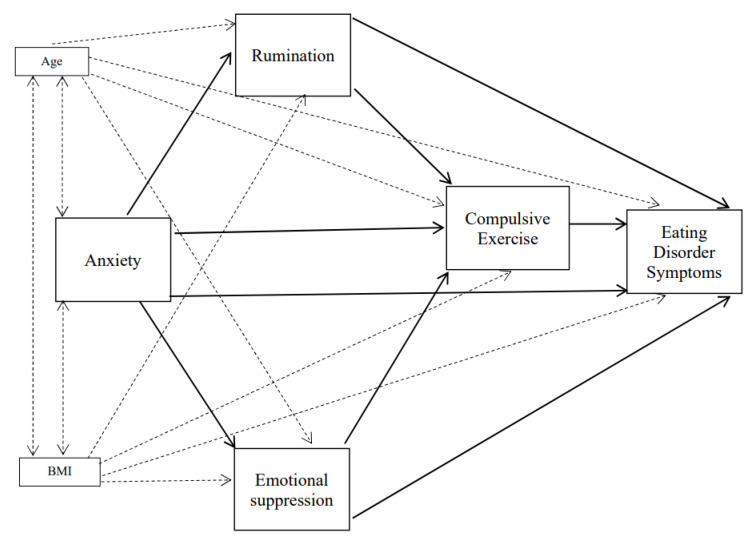
Initial Model (Body mass index (BMI) and age were entered as covariates in the model). Dotted lines represent the link between the covariates and the variables in the model. Solid lines refer to the direct links between the constructs in the model.

A logical next step for research in this area, therefore, is to test whether rumination and emotional suppression mediate the association between anxiety and compulsive exercise in the prediction of eating disorder symptoms in adolescents (see Figure 1). First, we predicted that rumination (Hypothesis 1) and emotional suppression (Hypothesis 2) were significantly and directly associated with compulsive exercise. Secondly, in the context of the interacting cognitive processes suggested by the ECM, we expected significant effects of anxiety on compulsive exercise to occur through rumination and emotional suppression (Hypothesis 3). Thirdly, in line with the compulsive exercise model [1], we predicted that compulsive exercise would be related to greater ED symptoms (Hypothesis 4). In adolescents, evidence suggests that body mass index (BMI) may be a risk factor for eating disorder symptoms [51,52], therefore, BMI was introduced as covariate in the model.

## 2. Materials and Methods

### 2.1. Participants

A total of 212 adolescent males (M = 13.39, SD = 1.22 years) and 189 adolescent females (M = 13.64, SD = 1.29 years) aged from 12 to 17 years-old, recruited from 8 secondary schools in Castilla-La Mancha (Spain) participated on the study. All participants were studying the compulsory secondary education in the Spanish education system. Inclusion criteria involved an age between 11 and 18 years; to understand the self-reported questionnaires; to give informed consent; and to have the written consent of their parents/legal guardians to participate in the study.

### 2.2. Instruments

Eating disorder symptoms were assessed with the Spanish version of the Eating Disorder Inventory-3 (EDI-3) [53,54]. The 25-item EDI-3 consists of three subscales. The Drive for Thinness subscale (DT-EDI-3) examines the desire to be thinner and explores concerns about food and weight. The Bulimia subscale (B-EDI-3) measures aspects related to binge eating and the extent to which food intake is linked to negative emotional states. The Body Dissatisfaction subscale (BD-EDI-3) assesses the extent of dissatisfaction with body shape and size. Items are rated on a 6-point Likert-scale (0 = Never; 5 = Always). The EDI-3 showed good internal consistency (α = 0.86) in this sample.

The 7-item State-Trait Anxiety Inventory-Trait subscale (STAI-A) [55] from the State-Trait Anxiety Inventory (STAI) [56] was used. The STAI-A subscale assesses dispositional anxiety. Items were rated on a 4-point Likert-scale (0 = almost never; 3 = almost always). This subscale showed good internal consistency in the current sample (α = 0.72).

To measure compulsive exercise, the Spanish version of the Compulsive Exercise Test (CET) [57,58] was used. The CET is comprised of 24 items over 5 subscales: (a) Avoidance and Rule-Driven Behaviour; (b) Lack of Exercise Enjoyment; (c) Weight Control Exercise; (d) Mood Improvement; and (e) Exercise Rigidity. The CET is answered using a 5-point Likert-scale (0 = never true; 5 = always true). Higher scores are indicative of higher levels of compulsive exercise. A recent study with Spanish adolescents showed a good model fit of the CET when items 5 and 8 were removed [59], hence these items were also removed in this study. In this sample, the overall internal consistency of the CET was good (α = 0.88).

Rumination was measured with the Spanish adaptation of the Short Depressive Rumination Scale (SDRS) [60,61]. The SDRS consists of four statements derived from the Leuven Adaptation of the Rumination on Sadness Scale (LARSS) [62]. The scale evaluates rumination frequency in sad, down or depressed situations on a 10-point Likert scale (0 = not at all, 10 = very often). The internal consistency of the SDRS was good in the current sample (α = 0.83). 

Emotional suppression was assessed via the expressive suppression subscale of the Emotion Regulation Questionnaire (ERQ) [63]. The ERQ was adapted to Spanish by Cabello et al. [64] and validated in an adolescent sample by Gómez-Ortiz and colleagues [65]. This subscale is composed of 4 items which require a response on a 7-point Likert scale (1 = totally disagree, 7 = totally agree). The reliability of this subscale was found to be good in this sample (α = 0.71).

Body Mass Index (BMI) was assessed by measuring participants’ height and weight, following the standardizsed protocol by the International Society for the Advancement of Kinanthropometry (ISAK) [66]. Height was measured using a portable height measure (Tanita, HR001) with a graduation of 1 mm. Weight was measured using a calibrated digital scale (Tanita, model HD-366) with a sensitivity of 0.1 kg.

### 2.3. Procedure

This study was approved by the Albacete Hospital Ethics Committee. Adolescents and their parents were provided with details and the aims of the study via an information letter. The information letter also explained that participation in the study was voluntary and that participants could withdraw from the research at any time, without any repercussions. Finally, parents were provided with a telephone number and an email address to contact if they had any concerns about the research. Participants (and their parents) who agreed to participate provided written consent. Completion of the questionnaires occurred over two sessions (both of which took 15 to 30 min). A total of 28 participants were eliminated from the results because they were absent from school when the data collection was conducted. The percentage of participants with missing data was very low (2.35%). None of the questionnaires with missing data contained more than three missing values. Hence, to handle missing data, missing items were replaced with the median of that item from the rest of the sample. An author of the research was present at all data collection sessions in order to answer any questions or concerns. Before answering the questionnaires, the research assistant explained the main aims of the study and reminded participants of the possibility to withdraw without any consequences. At the end of the second session, the weight and height of each participant were individually measured by two researchers in a quiet room. To reduce bias in the weight measurement, participants were asked to wear shorts and a sleeveless T-shirt in the second session. We subtracted 0.5 kg (approximate weight of long trousers) from the weight of participants who wore long trousers (*n* = 161) instead of shorts at the second session, to obtain a more accurate weight.

### 2.4. Statistical Analysis

First, Spearman’s correlations, means and standard deviations were calculated using SPSS v.23. Mann-Whitney U test was used to examine if there were significant differences between males and females on the measures. Second, in order to test our main aim (the functioning of compulsive exercise in the ECM prediction of EDs) and analyses direct effects between variables (Hypothesis 1 and 2), structural equation models (SEMs) were evaluated using the AMOS 19.0 software. The maximum likelihood was used to estimate all model parameters. In order to evaluate the fit of the models, we used χ2 statistic, the Comparative Fit Index (CFI) [67,68], and the root-mean-square error of approximation (RMSEA) [69]. According to Bentler [67], CFI values greater or equal to 0.90 are indicative of an acceptable fit. With regards to RMSEA, values lower or equal to 0.08 represent a reasonable fit [70]. With regards to χ2, a non-significant χ2 has been considered indicative of good fit [70].

Third, the indirect effects of anxiety on compulsive exercise via rumination and emotional suppression (Hypothesis 3) were examined with the bootstrapping sampling procedure [71]. Bias-corrected 95% confidence intervals on 5000 bootstrap samples were estimated for all direct and indirect effects. If the confidence interval did not include zero, the effect was significant.

Finally, post hoc power analyses were conducted following Jobst et al.’s specifications [72]. According to this procedure, the sample size used, the RMSEA model value, the α error probability and the model degrees of freedom are required to compute the statistical power of the tested model. For the analysis, we used the R package semPower [73]. Post hoc power values for the RMSEA statistic were calculated separately for males and females. For each group, the α error probability was specified to 0.050.

## 3. Results

### 3.1. Sample Characteristics

The CET, EDI and emotional suppression scores represent average to low levels of compulsive exercise, eating symptoms and emotional suppression for this age group [15,74]. Overall rumination levels were similar to other studies [60].

### 3.2. Descriptive Statistics and Correlations

Table 1 shows descriptive statistics (Means and SDs) and Spearman’s correlations for the variables of anxiety (STAI-A), rumination (VARS), emotional suppression, compulsive exercise (CET) and ED symptoms (EDI-3). Compulsive exercise significantly and positively correlated with anxiety, rumination and the total ED symptoms score in both males and females. Compulsive exercise was significantly and positively associated with emotional suppression in females, but not males. Age was not significantly linked to any of the variables in males. However, in females, age was significantly and positively related to anxiety and ED symptoms. BMI was significantly and positively associated with compulsive exercise and ED symptoms in both males and females.

Finally, Mann-Whitney U tests (Table 1) showed that the levels of anxiety, rumination and eating disorder symptoms were significantly higher in females compared to males.

### 3.3. Structural Equation Models

**Figure 2:**
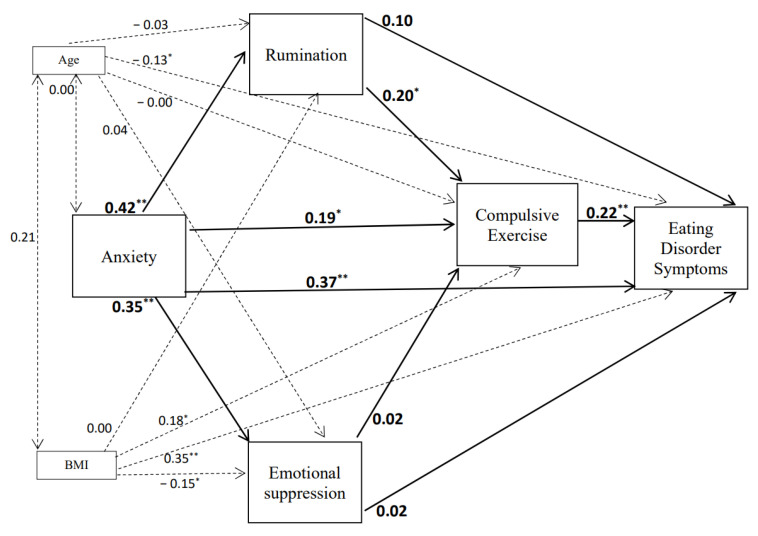
Final Structural Equation Model for males. Standardised parameters of direct effects are presented. Dotted lines represent the link between the covariates and the variables in the model. Solid lines refer to the direct links between the constructs in the model; * *p* < 0.05; ** *p* < 0.001.

**Figure 3:**
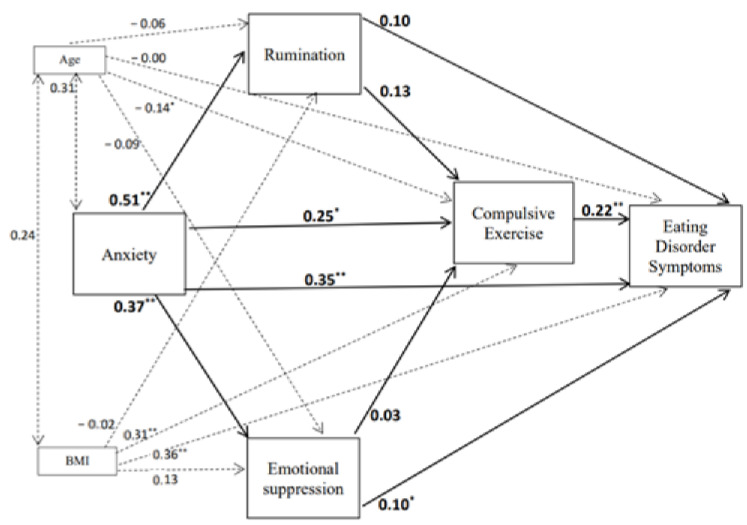
Final Structural Equation Model for females. Standardised parameters of direct effects are presented. Dotted lines represent the link between the covariates and the variables in the model. Solid lines refer to the direct links between the constructs in the model; * *p* < 0.05; ** *p* < 0.001.

The emotional cascade model with compulsive exercise added as a dysfunctional emotional behaviour that was initially tested is shown in Figure 1. The fit adjustment of the hypothesised emotional cascade model was good in females (χ2(1) = 0.54, *p* = 0.464, CFI = 1.00, RMSEA < 0.001) but, in the male group, the fit was not adequate (χ2(1) = 4.91, *p* = 0.027, CFI = 0.98, RMSEA = 0.14). Therefore, given that BMI was not significantly associated with anxiety levels in females or males, we removed the direct effects between BMI and anxiety in the models. These new models provided a good fit for males (χ2(2) = 2.58, *p* = 0.07; CFI = 0.98; RMSEA = 0.08) and females (χ2(2) = 1.24, *p* = 0.28; CFI = 0.99; RMSEA = 0.03). Figure 2 and Figure 3 show the standardised parameters of the models for males and females, respectively. According to these models, anxiety and compulsive exercise scores make the most significant contributions in explaining scores on the EDI-3, adjusting for BMI and age, in both males and females. Anxiety was also found to predict rumination, emotional suppression and compulsive exercise scores in both males and females. Whilst compulsive exercise was also predicted by rumination scores in males, the association between rumination and compulsive exercise in females was not significant.

In males, with regard to indirect effects in the model, when covarying for BMI and age, results showed that anxiety had significant indirect effects on compulsive exercise (standardised indirect effect = 0.09 [BC 95% CI = 0.01–0.18], *p* = 0.018) and eating disorder symptoms (standardised indirect effect = 0.12 [BC 95% CI = 0.05–0.19], *p* = 0.001). Rumination was also found to have a significant indirect effect on eating disorders symptoms via compulsive exercise (standardised indirect effect = 0.04 [BC 95% CI = 0.00–0.10], *p* = 0.01), adjusting for BMI and age. However, emotional suppression did not have significant indirect associations with compulsive exercise or eating disorder symptoms.

In females, the results showed that anxiety had a significant indirect effect on eating disorder symptoms (standardised indirect effect = 0.17 [BC 95% CI = 0.10–0.26], *p* < 0.001) when adjusting for BMI and age. However, anxiety had a non-significant indirect effect on compulsive exercise (standardised indirect effect = 0.08 [BC 95% CI = −0.01–0.18], *p* = 0.101), adjusting for BMI and age. Finally, neither rumination nor emotional suppression had a significant indirect effect on eating disorder symptoms.

To assess whether there were variables in the model that were differently related with regards to gender, a multigroup comparison was calculated. Thus, the test of gender equivalence involved the comparison of a model in which the structural weights and covariations among the weights were allowed to vary across gender groups (unconstrained model) to a model in which the weights and their covariations were constrained to be equal across the gender groups. The models were compared using a standard “decrement to $\chi^{2}$ test, in which the respective goodness of fit (and degrees of freedom) of the two models was differenced. The comparison of the unconstrained model ($\chi^{2}$(4) = 7.67, *p* = 0.105) with the constrained model ($\chi^{2}\left(21\right)=30.67,\;p=0.079$) was not significant ($\Delta \chi^{2}$ = 23.00, Δdf = 17, *p* = 0.14). This indicated that the model was consistent across gender groups.

Finally, post hoc model power calculated for the RMSEA statistic for the male sample (*n* = 212; RMSEA = 0.08; df = 2) and for the female group (*n* = 189; RMSEA = 0.03; df= 2) were 0.66 and 0.91, respectively.

## 4. Discussion

Compulsive exercise is considered a key component of EDs [1]. However, as far as we are aware, the roles of rumination and emotional suppression as mediators in the relationship between anxiety and compulsive exercise have yet to be tested.

Our first hypothesis, that rumination would be significantly and directly linked to compulsive exercise, was confirmed in males but not in females. The significant link between rumination and compulsive exercise suggests that rumination may have an important role on harmful attitudes and behaviours towards exercise in adolescent males. Rumination has been suggested a common underlying mechanism for dysfunctional behaviours [17,18,20] and a transdiagnostic feature across psychopathology [27,75]. A number of psychopathological mechanisms that involve compulsive exercise (e.g., affect, perfectionism, eating psychopathology and obsessive-compulsiveness) are related with maladaptive repetitive thoughts [27,28,29,30,31], so it is plausible to suggest that, rumination as a transdiagnostic feature, could also contribute towards the maintenance of exercise for mood, rigid exercise schedules, perfectionistic and obsessive-compulsive attitudes towards exercise. However, given the cross-sectional nature of this study, it is also plausible to suggest that compulsive exercise may increase the risk of rumination. Therefore, further research exploring the temporal links between rumination and compulsive exercise is needed.

In males, another interesting finding of the present study was that, while rumination had an indirect effect on ED symptoms through compulsive exercise, rumination did not have significant direct effects on ED symptoms. These results suggest that harmful attitudes and cognitions towards exercise may mediate the relationship between both rumination and ED symptoms. This hypothesis is in line with some studies that suggest that exercise could be the first symptom to appear in EDs, before the onset of a restrictive diet [3,76,77]. Therefore, exercise could serve as a “gateway” towards eating psychopathology, leading youths to utilise other more harmful compensatory behaviours if it is left unchecked [77,78]. However, this interpretation should be taken with caution, as this is a cross-sectional study, and therefore, causal relationships cannot be established.

In females, however, contrary to our first hypothesis, rumination was not directly linked to compulsive exercise. This result is in line with the findings of Smith and colleagues [32], in that rumination did not emerge as significant predictor for compulsive exercise. The lack of significant effects between rumination and compulsive exercise in females could be due to the cognitive content of rumination that has been examined in this study (depressive rumination). Evidence has suggested that rumination may have a specific function in individuals with EDs (focused on preoccupations with details of planning, calorie counting, eating, and exercise schedules), which differs qualitatively from depressive rumination [29,79,80]. Moreover, in a recent meta-analysis, Smith et al. [29], found that the association between ED-specific rumination and ED symptoms was stronger than the relationship between general rumination and ED symptoms. But to our knowledge, eating disorder-specific rumination has not yet been investigated in relation to compulsive exercise. Likewise, thoughts focusing on concerns about muscularity (e.g., muscle size and shape, exercise to increase muscles) could increase the risk of compulsive exercise, drive for muscularity and ED symptoms in adolescents. For this reason, future longitudinal and experimental studies focused on the role and content of ruminative thoughts could be helpful to understand which thoughts are the most harmful and that underlie compulsive exercise.

Contrary to our second hypothesis, emotional suppression was not significantly and directly associated with compulsive exercise neither in males nor females. The lack of relationships between emotional suppression and compulsive exercise is in line with other studies [32]. However, the findings from this study showed that emotional suppression had an indirect effect on ED symptoms through compulsive exercise among females but not in males, suggesting in line with outcomes in females with EDs [38], that compulsive e exercise may be used as strategy to replace emotional verbal expression and reduce emotional discomfort. 

Our third hypothesis, that anxiety has indirect effects on compulsive exercise through rumination and emotional suppression, was confirmed only in males. Supporting the ECM [17], these results suggest that emotional cascades generated by the interaction between anxiety, rumination and emotional suppression may increase the risk for compulsive exercise in males. These findings highlight the importance of further longitudinal and experimental research to unpack the temporal links between emotional cascades and harmful exercise attitudes. However, it is noteworthy that, in both males and females, anxiety had a significant indirect association with eating disorder symptoms, suggesting that anxiety, emotion dysregulation (rumination and emotional suppression) and harmful exercise attitudes and behaviours may interact in the contribution of eating disorder symptoms. These results support previous evidence regarding the ECM in eating disorder symptoms, suggesting a key role of emotional dysregulation in eating disorder symptoms in adolescence [17,20]. Finally, in line with the compulsive exercise model [1], our findings also confirmed our fourth hypothesis that compulsive exercise would be associated to other eating disordered cognitions and behaviours, in both males and females. 

The findings have practical implications for research and prevention. The results from this study suggest that the inclusion of strategies to reduce rumination in preventative programmes could help to reduce the risk of some dysfunctional behaviours [81], specifically, compulsive exercise in males and eating disorder symptoms in both males and females. Findings from recent randomised controlled trials had shown that interventions focused on reducing the tendency to engage in repetitive negative thoughts significantly decreased symptoms of anxiety and depression in adolescents and young adults [82,83]. Therefore, targeting transdiagnostic risk factors, such as rumination, may be a promising approach for prevention [83], including not only anxiety and depression, but also for reducing harmful exercise and eating attitudes and behaviors. 

This study has some limitations to consider. First, ED symptoms were assessed through self-report. Second, instead of assessing only the frequency of exercise as a dysfunctional emotional behaviour, attitudes and behaviours towards exercise were measured. The main reason for doing this was that the frequency of exercise is not an adequate measure of assessment to distinguish between clinical and non-clinical populations [84]. Individuals could practise high frequency, intensity or duration of physical activity for reasons that are not related to the body, such as enjoyment or health [85]. However, exercise cognitions provide a better characterization of exercise in EDs [86,87]. Indeed, Danielsen et al. [87] found that exercise in terms of frequency, duration and intensity were not associated with ED symptoms in males with and without EDs. In future studies with clinical populations, it could be interesting to analyse not only exercise cognitions, but also physical activity frequency, motor restlessness and hyperactivity. Third, given the model power was below 0.8 in males, results from this group should be interpreted with caution. Fourthly, depression levels were not measured in this study, which could account for some of the discrepancies with previous findings. Finally, further explorations of the role of compulsive exercise for predicting specific ED symptoms (e.g., restraint, bulimic symptomatology, body dissatisfaction), beyond the overall ED symptoms that were considered in this study.

## 5. Conclusions

These preliminary results suggest that rumination (and not emotional suppression) may mediate the relationship between anxiety and compulsive exercise among male adolescents. These results are in line with the ECM, and suggest that harmful attitudes and behaviours may be used to cope with anxiety and rumination. Compulsive exercise, in turn, may be a dysfunctional emotional behaviour that also mediates the association between rumination and eating disorder symptomatology in adolescent males. Further experimental and longitudinal studies are needed to verify our findings and test compulsive exercise as a dysfunctional behaviour in the ECM. These findings highlight the importance of analysing emotional regulation strategies separately in order to understand the role of emotion regulation in ED symptomatology and improve preventive and treatment programs.

## Figures and Tables

**Table 1:** Descriptive statistics, bivariate correlations between anxiety, rumination, compulsive exercise and ED symptoms, and gender differences.

	(1)	(2)	(3)	(4)	(5)	(6)	(7)	M (Males)	SD (Males)	U
(1) STAI-A ^1^		0.41 **	0.34 **	0.29 **	0.49 **	0.04	0.05	70.21	30.86	14,144 **
(2) SDRS ^2^	0.48 **		0.27 **	0.26 **	0.34 **	−0.00	0.02	140.28	90.40	14,012 **
(3) ES-ERQ ^3^	0.36 **	0.21 **		0.11	0.20 **	0.02	−0.09	130.84	50.46	19,785
(4) Total CET ^4^	0.30 **	0.21 **	0.18 *		0.41 **	0.05	0.19 **	100.09	30.57	18,720
(5) Total EDI-3 ^5^	0.51 **	0.35 **	0.33 **	0.52 **		−0.00	0.33 **	150.31	120.88	15,641
(6) Age	0.34 **	0.09	0.08	0.03	0.23 **		0.25 **	130.39	10.22	17,914 **
(7) BMI ^6^	0.11	−0.01	0.10	0.33 **	0.51 **	0.24 **		200.61	30.28	19,298
**M (females)**	90.60	190.23	140.07	90.67	210.43	130.64	200.52			
**SD (females)**	40.76	90.59	50.41	30.58	160.74	10.29	30.66			

Adolescent males above and adolescent females below. ^1^ STAI-A = Trait subscale of the State-Trait Anxiety Inventory; ^2^ SDRS = Short Depressive Rumination Scale; ^3^ ES-ERQ = Emotional Suppression from the Emotion Regulation Questionnaire; ^4^ CET = Compulsive Exercise Test; ^5^ EDI-3 = Eating Disorder Inventory-3; ^6^ BMI = Body Mass Index * *p* < 0.05; ** *p* < 0.001.

## Data Availability

Not applicable.
